# Engrailed homeobox 1 transcriptional regulation of COL22A1 inhibits nasopharyngeal carcinoma cell senescence through the G1/S phase arrest

**DOI:** 10.1111/jcmm.17575

**Published:** 2022-10-05

**Authors:** Mao‐Ling Huang, Wen‐Long Luo

**Affiliations:** ^1^ Department of Otorhinolaryngology Head and Neck Surgery The Second Affiliated Hospital of Chongqing Medical University Chongqing China

**Keywords:** cell cycle, cell senescence, NPC, the CDK4/6‐cyclin D1‐Rb signalling pathway

## Abstract

EN1 is well known as a transcription factor in other tumours, but its role in NPC is unclear. In this study, we first used bioinformatics to analyse GEO data to obtain the differentially expressed gene EN1, and subsequently verified that EN1 was highly expressed in nasopharyngeal carcinoma cells by tissue microarrays as well as cell lines. Further, we down‐regulated the expression of EN1 in cells for RNA sequencing. The analysis of sequencing results using KEGG and GO revealed significant changes in cell proliferation and cycle function after downregulation of EN1. Meanwhile, we found that cells underwent senescence after inhibition of EN1 under electron microscopy and the SA‐β‐gal assays. Based on the sequencing results, we verified that EN1 can promote the proliferation and cycle of NPC cells in cell function experiments and animal experiments. To investigate how EN1 affects cell senescence, we found that EN1 transcriptional regulation of COL22A1 regulated cell proliferation and cycle via CDK4/6‐cyclin D1‐Rb signalling pathway by dual luciferase reporter, Immunoblotting and rescue experiment. Accordingly, we uncovered that EN1 could serve as a target for the regulation of senescence in NPC.

**Abbreviations**
COL22A1: Collagen type XXII alpha 1 chain
DEGs: Differentially expressed genes
EN1: Engrailed homeobox 1
NPC: Nasopharyngeal carcinoma
NPG: Nasopharyngitis

## 1 INTRODUCTION

Nasopharyngeal carcinoma (NPC) is a malignant tumour of the head and neck that is highly prevalent in the south and southeast coastal areas of China, and about 80% of NPC in the world occur in China.^1^ It is now believed that the development of nasopharyngeal carcinoma is a combination of genetic, EBV infection and environmental factors.^2^ Due to its insidious location (pharyngeal fossa) and rapid progression, it is often accompanied by metastasis of lymph nodes when first detected and is prone to recurrence after treatment along with a poor prognosis.^3^ Researchers have been actively exploring the mechanisms underlying the development and metastasis of nasopharyngeal carcinoma. It was found that neoplastic spindle cell is valuable morphological predictor to account for tumour dissemination and metastasis in NPC.^4^, ^5^ Therefore, we need to explore and explain the mechanisms of nasopharyngeal carcinogenesis and metastasis in greater depth.

Senescence refers to a permanent cell cycle arrest triggered by a variety of stimuli. There has been considerable research on the role of replicative senescence, DNA damage‐induced senescence and oxidative stress‐induced senescence in tumour cell senescence.^6^ Replicative senescence, that is cell cycle arrest after multiple divisions resulting in reduced proliferative capacity. Huang et al.^7^ found that Coroglaucigenin induces senescence in colorectal cancer cells. Crochemore et al.^8^ found that CSB promoter downregulation via histone H3 hypoacetylation is an early determinant of replicative senescence. As a result of cellular senescence, cancer cells cannot proliferate irreversibly. Therefore, cellular senescence is a very important part of the anti‐tumour mechanism.

Engrailed homeobox 1 (EN1), is a member of the homeobox family. The human EN1 gene is located on chromosome 2q14.2. And the genomic DNA contains 7215 bases, which contains 2 exons. In recent studies, EN1 has been shown to play a significant role in tumorigenesis. For example, Guillermo et al.^9^ found that EN1 was highly expressed in triple‐negative breast cancer and that downregulation of EN1 inhibited the proliferation of breast cancer cells. Zhang et al.^10^ found that EN1 promotes the development of non‐small cell lung cancer by whole‐genome sequencing. Shunichi et al.^11^ identified EN1 as a possible diagnostic marker for adenoid cystic carcinoma and a diagnostic indicator for pleomorphic adenocarcinoma. It has not been reported that EN1 and NPC are correlated.

Collagen type XXII alpha 1 chain (COL22A1), a member of the collagen family. Most of the collagen members and collagen products are able to promote tumour development.^12^ The family member collagen XIII was found to inhibit anoikis and thus promote breast cancer progression.^13^ Collagen I was found to be closely associated with colon cancer progression by Wu et al.^14^ Only COL22A1 was found to be highly expressed in head and neck tumours,^15^ and no studies have been conducted to correlate COL22A1 with NPC.

We first obtained the differentially expressed gene EN1 by GEO data screening, and then down‐regulated EN1 in nasopharyngeal carcinoma cells followed by RNA sequencing revealed that the nasopharyngeal carcinoma cell cycle and the downstream gene COL22A1 were altered. Finally, we used functional experiments to verify these alterations.

## 2 METHODS

### 2.1 Bioinformatic Analysis

Differentially expressed genes (DEGs) in the GSE61218 dataset were analysed using |log2FC| ≥ 1 and adj. *p* < 0.05 as screening criteria.^16^ Validation of differential genes was performed using the GEPIA website. EN1 gene promoter were obtained from the UCSC database and EN1 downstream target gene was obtained from JASPAR database.

### 2.2 Cell culture and materials

Human nasopharyngeal squamous cell carcinoma cell lines (CNE2Z and SUNE‐1) and permanent nasopharyngeal epithelial cell lines (NP69) were obtained from the BLUEFBIO (CAT.NO.BFN60700251, CAT.NO.BFN60808628, CAT.NO.BFN60870097). Human nasopharyngeal squamous cell lines (5‐8F and HK‐1) were purchased from The World Cell Factory (NO.iCell‐h234, NO.iCell‐h367) and cultured in Roswell Park Memorial Institute (RPMI)‐1640 media (Biosun) with 10% fetal bovine serum (Gibco, Thermofisher) with a humidified atmosphere containing 5% CO2 at 37°C.^17^

Tissue microarrays (TMA, NO.132) were provided by Fanpu Biotech. NO.132 were used for EN1 analysis (Tables S1 and S2).

Palbociclib (PD‐0332991) HCl was purchased from SELLECK (CAS No.827022–32‐2).

### 2.3 Immunofluorescence assay

Tissue sections are blocked with 10% BSA for 1 h after baking, dewaxing, hydration and antigen recovery. The sections are then incubated with the antibody overnight. The next day, sections were incubated with FITC‐labelled or Cy3‐labelled antibodies for 1 h in a dark room. And subsequently sealed with anti‐fluorescence quenching solution, followed by observation and image acquisition under a fluorescence microscope.

### 2.4 Immunohistochemical staining analysis

Tissue samples were processed and analysed with reference to previous articles.^18^ The extent of EN1 staining was classified as negatively, weakly positively, moderately positively and strongly positively. Consequently, the EN1 low expression category was considered negative and weakly positive, while the EN1 high expression category was moderately positive and strongly positive.

### 2.5 Generation of knockdown or overexpressed cells

Cells were transfected with lentivirus treatment according to the previous article.^19^ Transfection of null‐loaded lentivirus tagged with shNC. and Cells transfected with pLKO.1‐puro‐EN1‐shRNA1 or pLKO.1‐puro‐EN1‐shRNA2 were labelled as shEN1‐1 or shEN1‐2. Cells transfected with pLV3‐CMV‐COL22A1 was labelled as oeCOL22A1 Cells transfected with plv2‐cmv‐en1(human)‐3 × flag‐puro was labelled as oeEN1.

Related plasmid profiles can be found in the (Figure S2).

### 2.6 qRT‐PCR assay

Refer to the previous literature for specific steps.^20^ The relative mRNA expression levels of target genes were calculated by the ${{2^{-}}^{\Delta \Delta }}^{C_{t}}$ method. The primer for EN1 was obtained from ORIGENE (CAT#: HP205309).

### 2.7 RNA‐seq data analysis

KEGG and GO analysis from public databases.^21^ ShNC group and shEN1 group cells were delivered to BIOMED (Chongqing) Co., Ltd. (boaimedicine.com) for high‐throughput transcriptome sequencing of multiple cell samples using the Illumina HiSeq sequencing platform. The specific steps for the analysis of the results can be found in the Bioinformatic Analysis section. Finally, the obtained DEGs were incorporated into the KEGG and GO database for further enrichment analysis.

### 2.8 Transmission electron microscopy

Cells were grown on six‐well plates; Trypsinization and 1X PBS were performed on them. We then collected the cells by centrifugation at 1000 *g* for 5 min and fixed them by resuspending them in 2.5% glutaraldehyde and incubating them for 2 h. Samples were fixed with osmium tetroxide in 0.1 M sodium cacodylate buffer, dehydrated through a graded series of acetone and then embedded in resin. To conclude, samples were cut into 65 nm‐thick sections and then processed for TEM. The TEM analysis was performed with a Hitachi H600 electron microscope (Hitachi, Ltd.).

### 2.9 Western blot analysis

For Western blot analysis, cells were cultured in 10 cm diameter dishes, and cells were collected after spreading over the dish. RIPA buffer supplemented with protease inhibitor cocktail (Cell Biolabs) was added to cells. Cells were broken by sonication on ice, and the supernatant was collected by centrifugation and added to the loading buffer. Subsequently, equal amounts of total protein (30–50 μg) per well were used for electrophoresis, and the membrane was transferred and closed after electrophoresis. Then, the primary antibody is incubated overnight, and the membrane is washed and incubated with secondary antibody after the incubation of primary antibody. Finally, the membranes were scanned. The antibodies used in the process were as follows: rabbit monoclonal EN1 antibody, dilution 1:500 (abcam, cat. no. ab 108598), rabbit polyclonal COL22A1 antibody, dilution 1:500 (abcam, cat. no. ab121846), rabbit monoclonal CDK4 antibody, dilution 1:1000 (abcam, cat. no. ab108357), rabbit monoclonal CDK6 antibody, dilution 1:1000 (abcam, cat. no. ab124821), rabbit monoclonal Cyclin D1 antibody, dilution 1:1000 (abcam, cat. no. ab16663), rabbit monoclonal p‐Rb (S807) antibody, dilution 1:1000 (abcam, cat. no. ab184796) and rabbit monoclonal GAPDH antibody.^22^

### 2.10 CCK‐8 and Colony formation assays

Cells (1 × 10e4) were grown into 96‐well culture plates and incubated for 24, 48 and 72 h. CCK‐8 (5 μl) was added to each well and incubated at 37°C for 30 min, followed by measurement of absorbance at 450 nm using SpectraMax iD5 (Molecular Devices).

Cells were inoculated into 6‐well plates at a rate of 500 per well, and after 14 days cells were stained with 0.1% crystal violet and photographed for counting.^22^

### 2.11 Flow cytometric analysis of the cell cycle

Cells were collected by centrifugation, washed once with 1 ml of PBS, added 500 μl PBS containing 50 μg/ml ethidium bromide (PI), 100 μg/ml RNase A and 0.2% Triton X‐100 and incubated for 30 min at 4°C, protected from light.^22^, ^23^ The data were analysed using FlowJo 7.6 software.

### 2.12 Construction of an NPC tumour model in nude mice

Four‐week‐old SPF‐grade BALB/C nude mice were selected for the experiment^24^ . The animal experiments were approved by the Committee on Ethics of Animal Experiments of Chongqing Medical University (NO. [2021]097. Date: 18 February 2121) and performed in compliance with the Guide for the Care and Use of Laboratory Animals from the National Institutes of Health. Nude mice were divided into two groups and subcutaneously injected with 0.1 ml of shNC or shEN1 cells, respectively. Daily observation and recording of tumour volume in each group. When the tumour size was about 1.5 cm^3^, the nude mice were executed and the tumours were taken for analysis. The final tumour volume (*V*) was calculated as follows^25^: *V* = (*a* × *b* × *b*)/2.

### 2.13 Luciferase reporter assay

The human EN1 gene was first inserted into the vector pcDNA3.1 (pCDNA3.1‐EN1). Subsequently, two promoter sequences of human COL22A1 were inserted into the vector pGL3‐Basic (labelled pGL3‐ COL22A1–1 and pGL3‐ COL22A1–2). The constructed plasmids and the pRL‐TK plasmid containing the Renilla (Sea Corydalis) luciferase sequence were transfected into 5‐8F and CNE‐2Z cells. Cells were grown to the appropriate density, then lysed, and the lysate was collected. Subsequently, firefly luciferase assay reagent and Renilla luciferase assay reagent were added to the machine for detection. Finally, the ratio of firefly luciferase intensity (RLU‐1) to Renilla luciferase intensity (i.e. RLU‐1/RLU‐2) was used as an internal reference for target gene activation in each sample to be assayed.^19^

### 2.14 Statistical Analysis

All data collected in this study were analysed using the medical statistics software spss 20.0, and the data are expressed as the mean ± standard deviation (^−^χ ± s) values. Comparisons of two independent samples were performed using a *t*‐test. The data of CCK‐8 assay and the growth curve of tumour in mice were performed using one‐way anova. GraphPad Prism 8 was used in this study to generate relevant statistical plots. For the purpose of this study, *p* < 0.05 was considered to indicate a statistically significant difference.

## 3 RESULTS

### 3.1 EN1 is highly expressed in nasopharyngeal carcinoma

**FIGURE 1:**
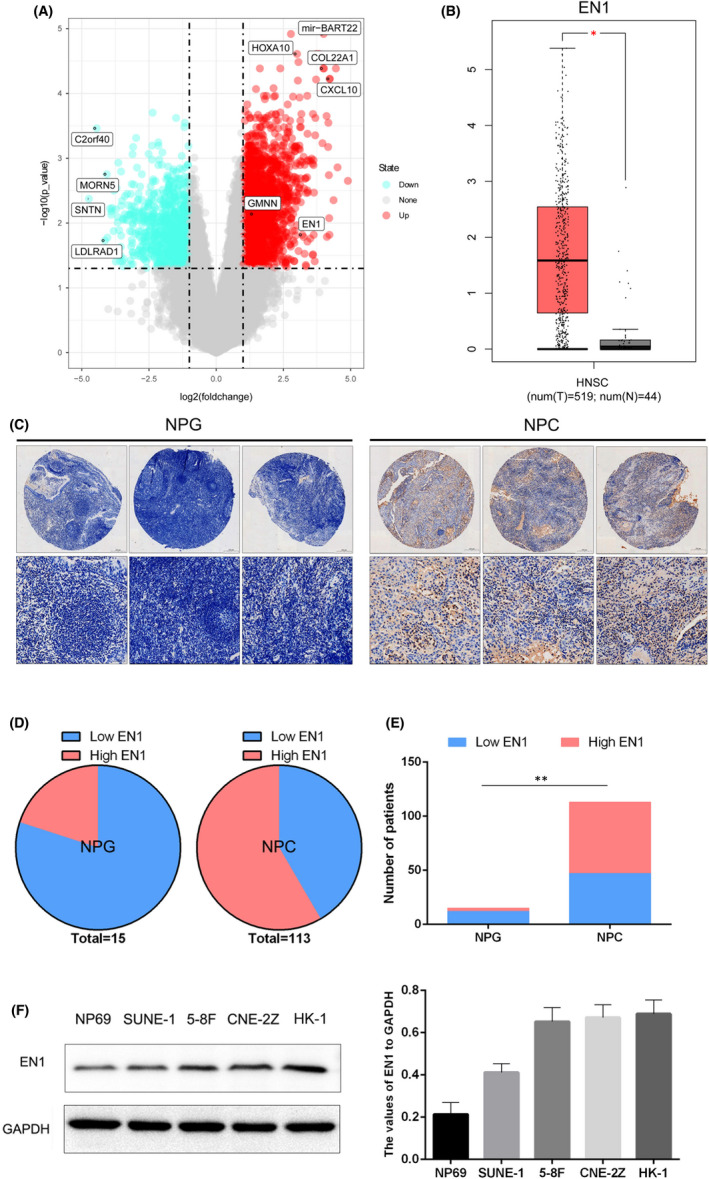
EN1 is highly expressed in NPC tissue cells. (A) Heat map of GEO data differentially expressed genes. (B) EN1 is highly expressed in TCGA‐HNSCC. (C) Expression of EN1 in nasopharyngeal carcinoma microarrays. (D) Sectoral statistics of EN1 expression in NPG and NPC. (E) Cardinality test for statistical EN1 expression in NPG and NPC. (F) Expression of EN1 in various cell lines of NPC. *p < 0.05, **p < 0.01

To understand the expression level of genes in nasopharyngeal carcinoma cells, we used bioinformatics to analyse the GEO database. Then, we found high expression of EN1 gene in nasopharyngeal carcinoma tissues using GEO data analysis (Figure 1A). Subsequently, we verified it using the TCGA database (Figure 1B). Further, tissue microarray results also showed high expression of EN1 in NPC tissues and low expression in NPG tissues (Figure 1C–E). Finally, the expression of EN1 in the cell lines NP69, SUNE‐1, 5‐8F, CNE‐2Z and 6‐10B was analysed. The results confirmed that CENP‐N was significantly high expression in several NPC cell lines (Figure 1F).

### 3.2 Downstream senescent pathways and functional changes in nasopharyngeal carcinoma cells after downregulation of EN1

**FIGURE 2:**
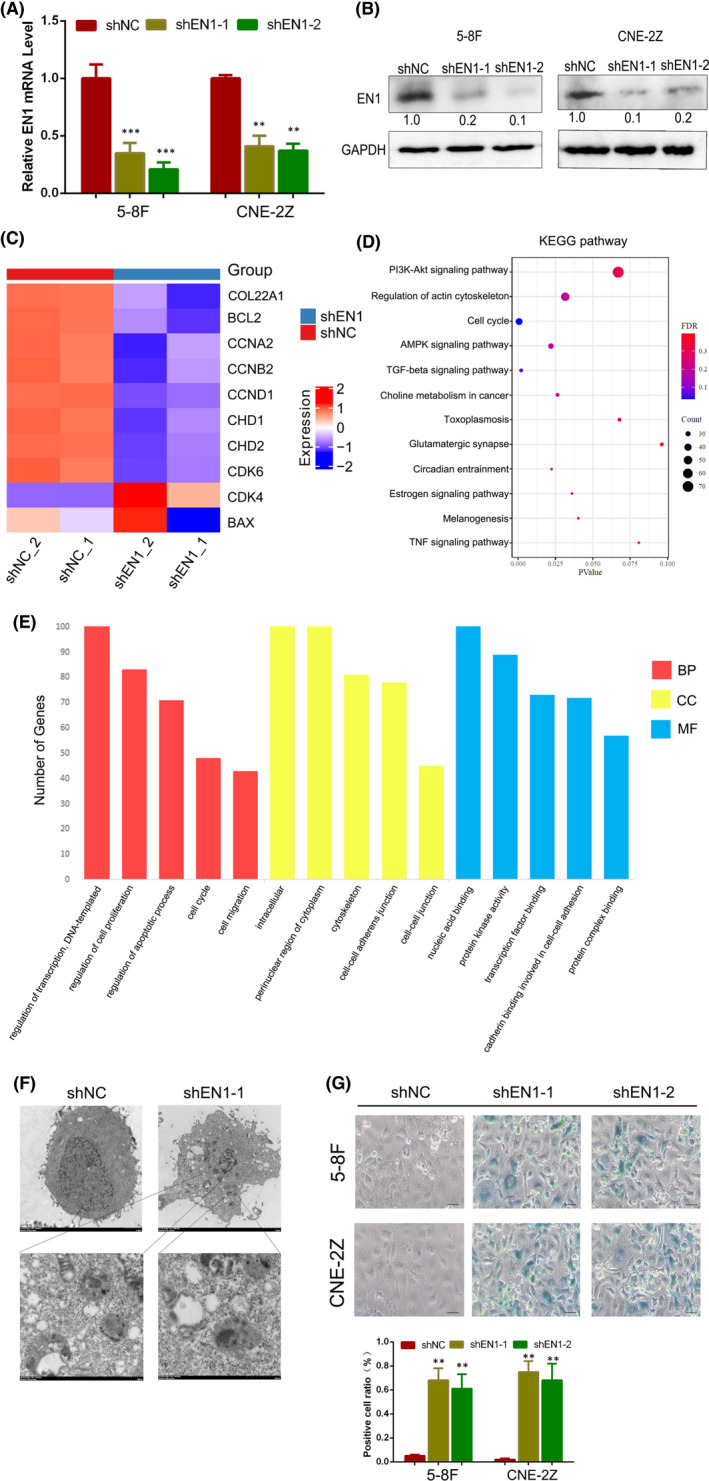
Changes in cell function after downregulating EN1. (A) Validation by qRT‐PCR after downregulation of EN1. (B) Verified by immunoblotting after downregulation of EN1 in 5‐8F and Cen‐2Z cell lines. (C) Heat map of the alteration of some genes after downregulation of EN1. (D) KEGG bubble diagram shows the function of EN1‐altered genes. (E) GO maps plotted for changes in genes after downregulation of EN1. (F) TEM showed many secondary lysosomes (including lipofuscin) appear in the shEN1 group. (G) the SA‐β‐gal assays showed a higher proportion of positive cells in the shEN1 group. The results show the mean ± SD from triplicate experiments. *p < 0.05, **p < 0.01, ***p < 0.001

To explore the function of EN1 in nasopharyngeal carcinoma cells, we down‐regulated EN1 in 5‐8F and CNE‐2Z cells (Figure 2A,B). Subsequently, we sequenced down‐regulated cells with control cells for RNA. It was found that downregulation of EN1 resulted in changes in downstream pathways mainly focusing on the regulation of cellular senescence (Figure 2C–E).

We observed under transmission electron microscopy that after inhibition of EN1, many secondary lysosomes (including lipofuscin) appeared in the cytoplasm of the cells, which indicated that senescence occurred (Figure 2F).

To verify the changes in the cellular senescence of nasopharyngeal carcinoma cells after downregulation of EN1, we performed cell function experiments. CCK‐8 assay showed that the proliferative capacity of the shEN1 group was significantly diminished compared with the shNC group (Figure S1). Colony formation assays showed that a decrease in single cell viability of the shEN1 group compared with the shNC group (Figure S1). Cell cycle assay showed that the proportion of G1 phase was increased in shEN1 compared with shNC (Figure S1).

To prevent the off‐target effect of RNAi, we performed rescue experiments on cells. CCK‐8 assay showed that the proliferative capacity of the shEN1 + oeEN1 group was significantly increased compared with the shEN1 group (Figure S2). Colony formation assays showed that an increase in single cell viability of the shEN1 + oeEN1 group compared with the shEN1 group (Figure S2). Cell cycle assay showed that the proportion of G1 phase was decreased in shEN1 + oeEN1 compared with shEN1 (Figure S2).

Combined with these results, we hypothesized that the replicative senescence was activated after downregulating EN1.

### 3.3 EN1 transcriptionally regulated COL22A1 and thus affected cell cycle‐related protein alterations

**FIGURE 3:**
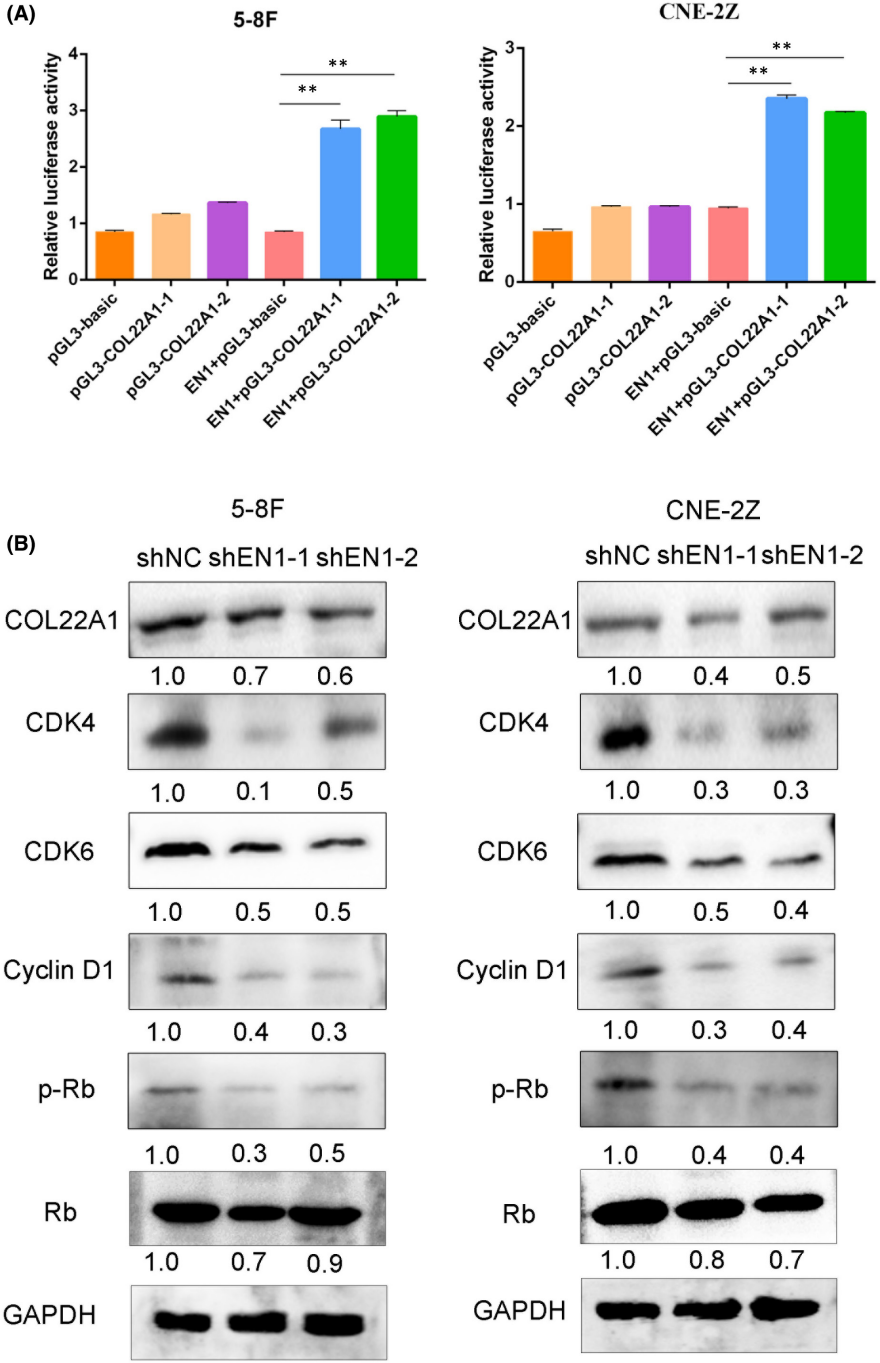
EN1 regulates cell proliferation and cycle via COL22A1‐CDK4/6‐Cyclin D1‐Rb signalling axis. (A) Dual luciferase reporter shows EN1 transcriptional regulation of COL22A1. (B) Immunoblotting experiments showed the expression of COL22A1, CDK4/6, Cyclin D1 and p‐Rb proteins. The results show the mean ± SD from triplicate experiments. *p < 0.05, **p < 0.01, ***p < 0.001.

To investigate how EN1 affects the nasopharyngeal carcinoma cell cycle, we verified the relationship between EN1 and COL22A1 using a dual luciferase reporter. The results showed that EN1 was able to transcriptionally bind the COL22A1 promoter sequence and promoted the transcription of COL22A1 (Figure 3A). Subsequently, to elucidate how EN1 affects cell cycle alterations, we explored downstream pathways. The results revealed that COL22A1, CDK4, CDK6, Cyclin D1 and P‐Rb protein levels were decreased after downregulation of EN1 (Figure 3B). This suggests that EN1 affects the cell cycle via the COL22A1‐CDK4/6‐Cyclin D1‐Rb signalling axis.

### 3.4 Overexpression of COL22A1 reversed the effect of downregulation of EN1 on the replicative senescence in NPC

**FIGURE 4:**
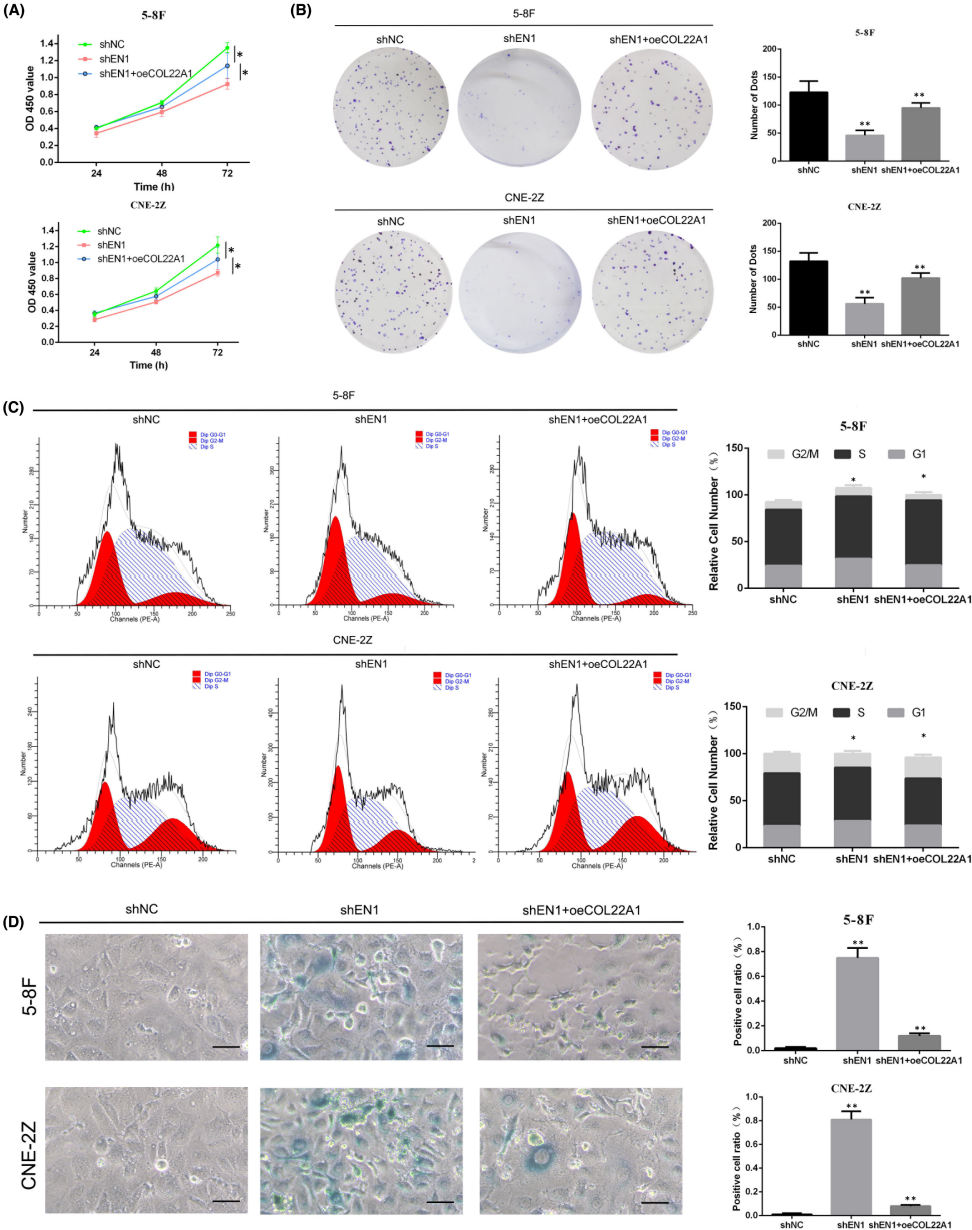
Overexpression of COL22A1 rescues cell proliferation and cycle changes caused by downregulation of EN1. (A) CCK‐8 assay detects cell proliferation capacity after simultaneous knockdown of EN1 and overexpression of COL22A1. (B) Single‐cell clone formation ability after simultaneous knockdown of EN1 and overexpression of COL22A1 in two NPC cell lines. (C) Flow cytometric cell cycle assay detects cell cycle changes in 5‐8F and CNE‐2Z cell lines. (D) The SA‐β‐gal assay detects proportion of positive cells in 5‐8F and CNE‐2Z cell lines. The results of CCK‐8 assay are analysed with one‐way anova. The other results are shown as mean ± SD. All data from triplicate experiments. *p < 0.05, ** *p* < 0.01, ***p < 0.001

To get a clearer picture of the upstream and downstream relationship between EN1 and COL22A1, we subjected NPC cells to downregulation of EN1 and upregulation of COL22A1. CCK‐8 assay showed accelerated growth of NPC cells in the shEN1 + oeCOL22A1 group compared to the shEN1 group (Figure 4A). Colony formation assay showed that the single cell cloning ability of NPC cells in the shEN1 + oeCOL22A1 group was increased compared to the shEN1 group (Figure 4B). Cell cycle assay showed that the proportion of G1 phase cells was reduced in the shEN1 + oeCOL22A1 group compared with the shEN1 group (Figure 4C).

### 3.5 CDK4/6 inhibitors reversed the effect of overexpression of COL22A1 on the replicative senescence in NPC

**FIGURE 5:**
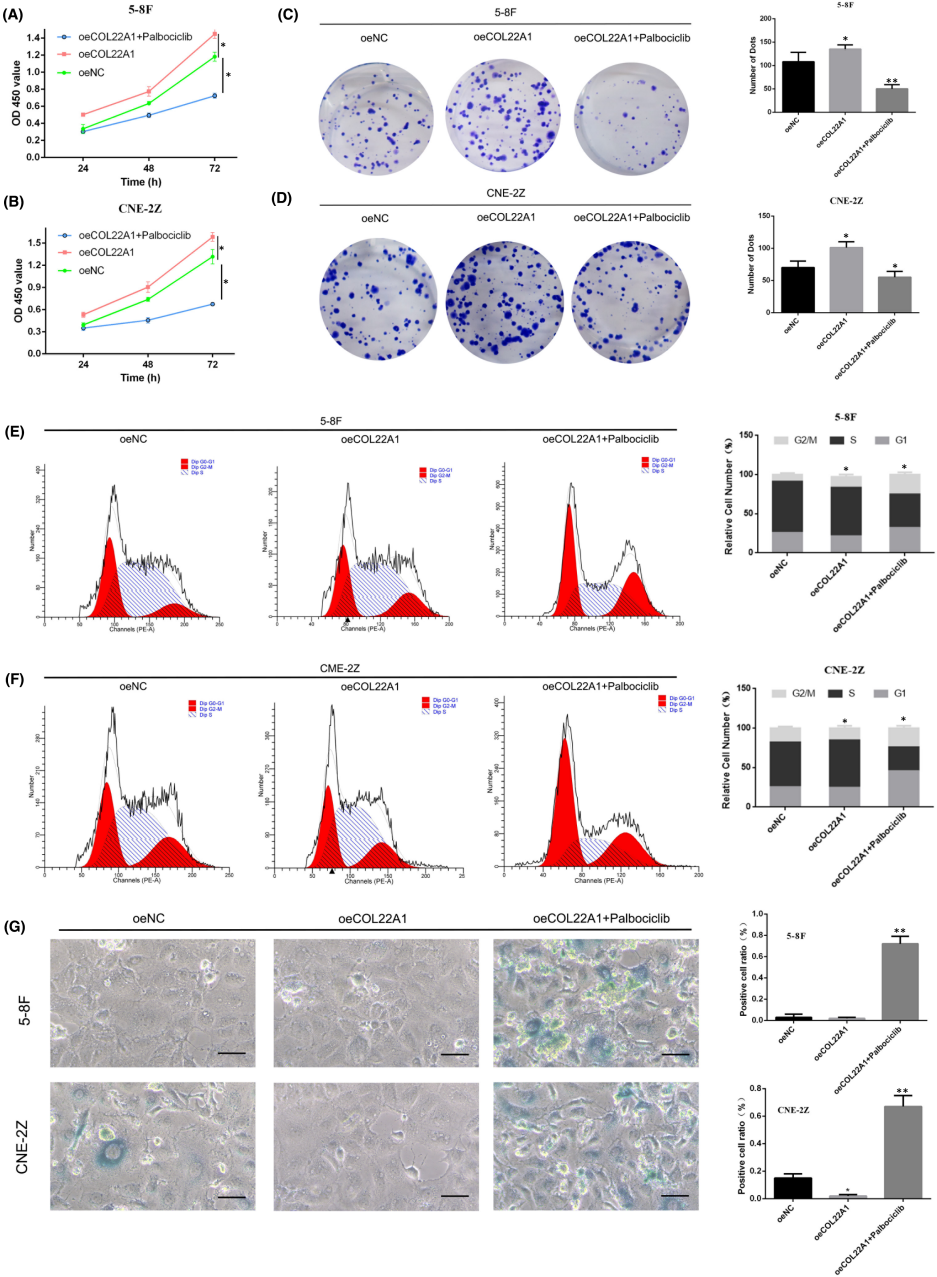
CDK4/6 inhibitors rescue cell proliferation and cycle changes caused by overexpression of COL22A1. (A, B) CCK‐8 assay detects cell proliferation capacity after simultaneous overexpression of COL22A1 and Palbociclib. (C, D) Single‐cell clone formation ability after overexpression of COL22A1 and Palbociclib in two NPC cell lines. (E, F) Flow cytometric cell cycle assay detects cell cycle changes in 5‐8F and CNE‐2Z cell lines. (G) The SA‐β‐gal assay detects the proportion of positive cells in 5‐8F and CNE‐2Z cell lines. The results of CCK‐8 assay are analysed with one‐way anova. The other results are shown as mean ± SD. All data from triplicate experiments. *p < 0.05, **p < 0.01, ***p < 0.001

To get the downstream pathways of COL22A1, we established overexpression of COL22A1 nasopharyngeal carcinoma cells with the addition of the CDK4/6 inhibitor (Palbociclib). CCK‐8 assay showed the growth of NPC cells was inhibited in the oeCOL22A1 + Palbociclib group compared with the oeCOL22A1 group (Figure 5A,B). Colony formation assay showed that the single cell cloning ability of NPC cells in the oeCOL22A1 + Palbociclib group was decreased compared with the oeCOL22A1 group (Figure 5C,D). Cell cycle assay showed that the proportion of G1 phase cells was blocked in the oeCOL22A1 + Palbociclib group was decreased compared with the oeCOL22A1 group (Figure 5E,F).

### 3.6 Effect of downregulated EN1 on cellular senescence in vivo

**FIGURE 6:**
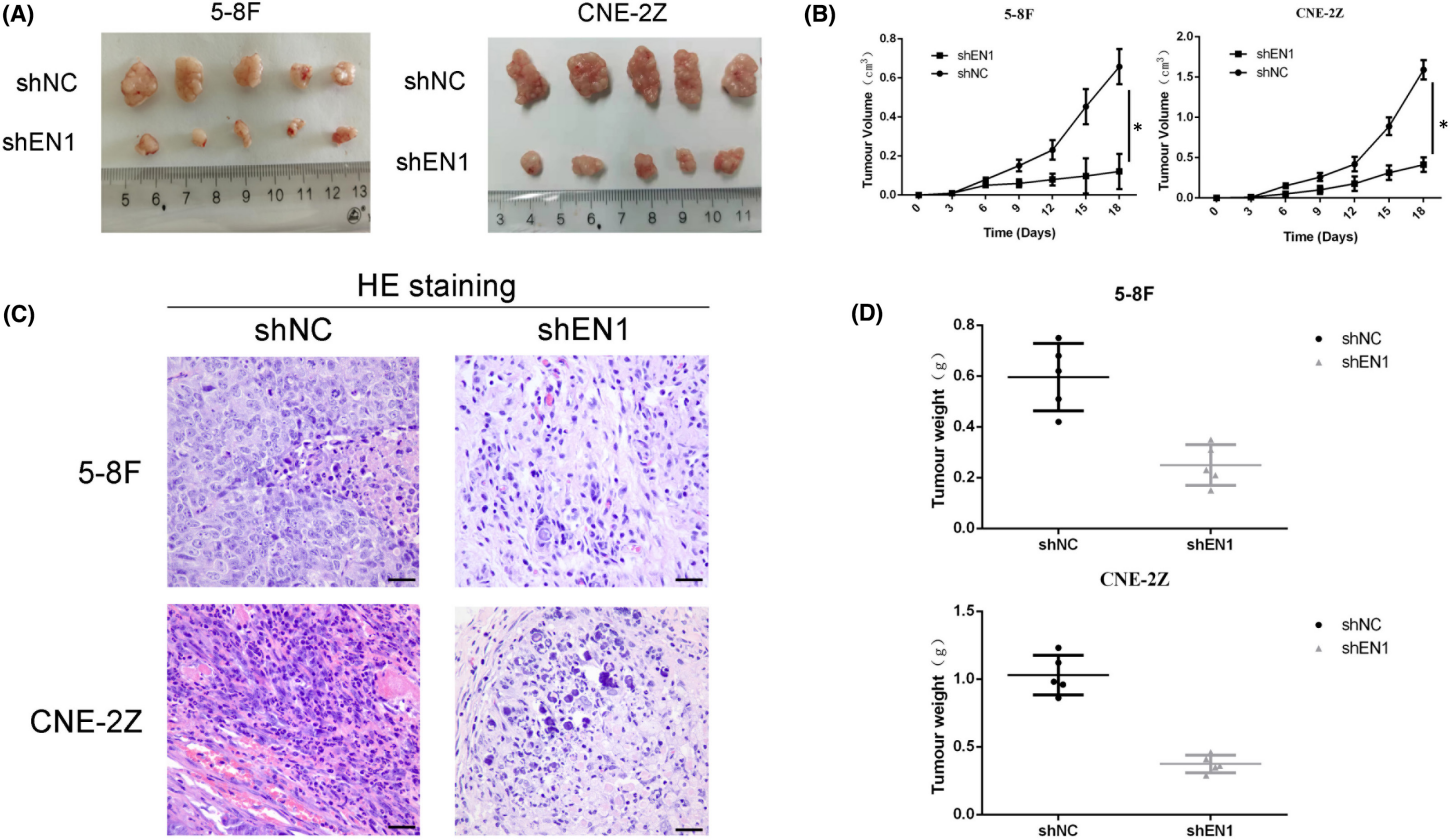
Downregulation of EN1 inhibited cell proliferation in vivo. (A) Overall view of the tumour after tumour formation in nude mice. (B) The growth curve of the tumour. (C) HE staining of the tumour. (D) Weight of the tumour. The results of the growth curve of the tumour are analysed with one‐way anova. *p < 0.05, **p < 0.01, ***p < 0.001

To further understand the effect of EN1 on cellular senescence in vivo, we established the lotus tumour model. Compared with the shNC group, the tumour volume in the shEN1 group was significantly reduced (Figure 6A). As shown in Figure 6B, the tumour growth curve of shEN1 group was significantly slower than that of shNC group. HE staining showed infiltration of tumour cells and inflammatory cells (Figure 6C). Compared with the shNC group, the tumour weight in the shEN1 group also decreased significantly (Figure 6D).

**FIGURE 7:**
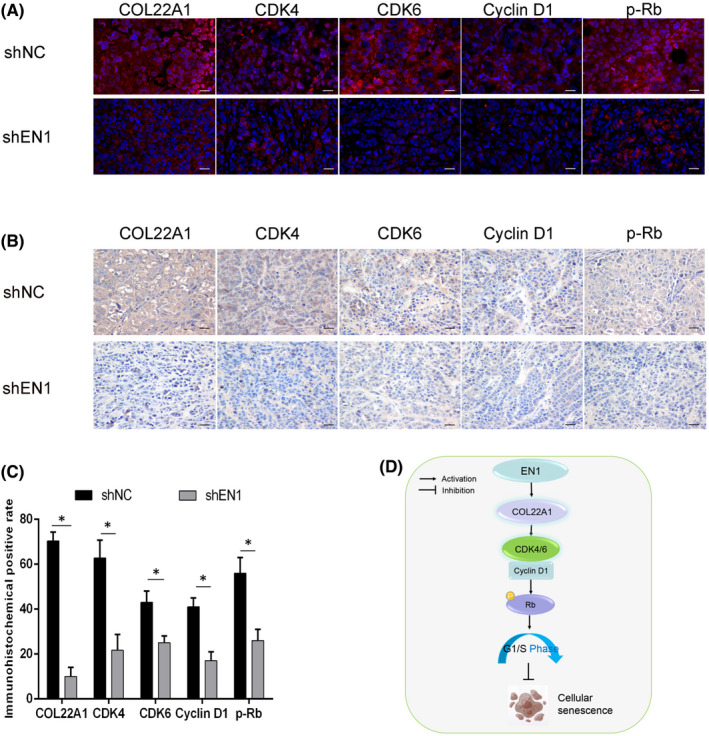
EN1 may regulate cell proliferation via COL22A1‐CDK4/6‐Cyclin D1‐Rb signalling axis in vivo. (A) Immunofluorescence showed the expression of COL22A1, CDK4/6, Cyclin D1 and p‐Rb proteins. (B) Immunohistochemistry showed the expression of COL22A1, CDK4/6, Cyclin D1 and p‐Rb proteins. (C) Statistical graph of immunohistochemistry. (D) Model diagram of EN1 action in NPC. Data are shown as mean ± SD. *p < 0.05, **p < 0.01, ***p < 0.001

Subsequently, we sectioned the tumours and performed immunohistochemistry and immunofluorescence staining. The results revealed that COL22A1, CDK4, CDK6, Cyclin D1 and P‐Rb protein levels were decreased after downregulation of EN1 (Figure 7A–C).

In summary, this study confirmed that the EN1‐COL22A1‐CDK4/6‐Cyclin D1‐Rb signalling axis can regulate the cellular senescence of nasopharyngeal carcinoma (Figure 7D).

## 4 DISCUSSION

The cellular senescence process occurs as a result of carcinogenic stress or oxidative stress. NPC can be effectively treated by inducing senescence in the cells.

In this study, we first used bioinformatics to analyse the GEO database of GSE61218 and found that EN1 was highly expressed in nasopharyngeal carcinoma. Subsequently, validation of EN1 using the TCGA‐HNSC database revealed that it was also highly expressed in head and neck tumours. Although the samples of the two databases are not identical, there is some predictive effect. To further determine the expression of EN1 in nasopharyngeal carcinoma tissues, we used nasopharyngeal carcinoma tissue microarrays as well as cell lines to find high expression of EN1 in nasopharyngeal carcinoma cells. These results are similar to those of Guillermo et al.^9^ who found high expression of EN1 in breast cancer.

To determine what effect EN1 has on the function of nasopharyngeal carcinoma cells, we performed cellular RNA sequencing after downregulating EN1. Through RNA sequencing, we are able to understand which functions and pathways are enriched by downstream altered genes.^26^ We used transmission electron microscopy to find an increase in secondary lysosomes in the cytoplasm of cells after inhibition of EN1 expression. And we found that the changes in cell proliferation and cycle were more obvious. Subsequently, we found that the proliferation capacity of nasopharyngeal carcinoma cells was reduced after downregulation of EN1, and the G1/S phase of cells was blocked using cell function experiments in vitro and in vivo.

RNA sequencing also revealed significant downregulation of COL22A1, and it has been minimally reported. We confirmed EN1 could bind directly to COL22A1 using a dual‐luciferase reporter and verified the relationship between EN1 and COL22A1 by rescue experiments. These results tentatively suggest that EN1 is capable of regulating the cell cycle through COL22A1.

The most typical feature of cellular senescence is cell cycle arrest. There is currently a wealth of clinical research and basic research on the cell cycle.^27^ Wang et al.^28^ found that Baicalin‐induced cellular senescence manifests as G1/S phase arrest. And Cell cycle inhibitors have been used in the treatment of many tumours, such as breast cancer, non‐small cell lung cancer (NSCLC), glioblastoma, melanoma and colorectal cancer.^29^ However, the treatment of tumours is increasingly limited because of the toxic effects of inhibitors and drug resistance.^30^, ^31^ Therefore, this requires a combination of more targeted drugs.

Cyclin‐dependent kinases (CDKs) and cyclin are the key point of action in cell cycle regulation.^32^ The main regulators involved in G1/S phase are CDK4/6 and Cyclin D1.^33^ Signalling stimulation promotes phosphorylation of Rb after CDK4/6‐Cyclin D1 complex formation, which completes the subsequent cycle process.^34^ Meanwhile, Zhou et al.^35^ found that lncRNA RP11‐624 L4.1 could regulate NPC cell proliferation through CDK4/6‐Cyclin D1‐Rb signalling axis. Therefore, we further found that EN1 transcriptional regulation of COL22A1 regulates cell proliferation and cycle via CDK4/6‐cyclin D1‐Rb signalling axis.

The regulation of cellular senescence is more complex. In this study, we only performed experiments around the EN1 transcriptional regulation of COL22A1 via the CDK4/6‐Cyclin D1‐Rb signalling axis and failed to systematically demonstrate the regulatory process of EN1. And the specific regulatory relationship between COL22A1 and CDK was not elucidated. We will further refine in future studies.

## 5 CONCLUSIONS

In summary, our results demonstrated that EN1 regulates cell proliferation and cycle in NPC. In addition, EN1 promotes the proliferation and cycle by transcriptional regulating COL22A1 via CDK4/6‐Cyclin D1‐Rb signalling pathway. These data highlight the importance of EN1 in the progression of NPC and revealed a novel target for further study.

## AUTHOR CONTRIBUTIONS

**Mao‐Ling Huang:** Funding acquisition (lead); writing – original draft (equal). **Wen‐Long Luo:** Writing – review and editing (equal).

## FUNDING INFORMATION

This work was supported by grants from Chongqing Postdoctoral Special Science Foundation (No.2010010005500683 and No.2010010005739004).

## CONFLICT OF INTEREST

The authors declare that they have no conflict of interest.

## Supporting information


Figure S1

Figure S2

Tables S1‐S2

Fig Legends S1‐S2

## Data Availability

The datasets used and/or analysed during the current study are available from the corresponding author on reasonable request.
