# Supplementary material for: Engrailed homeobox 1 transcriptional regulation of COL22A1 inhibits nasopharyngeal carcinoma cell senescence through the G1/S phase arrest
Source: J Cell Mol Med. 2022 Oct 5;26(21):5473–85. doi: 10.1111/jcmm.17575 (PMC9639036; doi:10.1111/jcmm.17575)
Supplement: Supplementary file 3 — Tables S1‐S2 [file JCMM-26-5473-s003.docx]

**Supplementary Table 1. General information of** **Tissue microarray**

| Characteristics | Type | Number |
| --- | --- | --- |
| Gender | male | 100 |
|  | female | 32 |
| Age(Y) | 50~ | 69 |
|  | 0-50 | 63 |

**Supplementary Table 2. Tissue microarray analysis of CENP-N expression**

| Group | CENP-N expression | | χ^2^ | P value |
| --- | --- | --- | --- | --- |
|  | Low | High |  |  |
| NPG | 12 | 3 | 8.533 | 0.0035 |
| NPC | 47 | 70 |  |  |

Note: **, P< 0.01.
