# Supplementary material for: Engrailed homeobox 1 transcriptional regulation of COL22A1 inhibits nasopharyngeal carcinoma cell senescence through the G1/S phase arrest
Source: J Cell Mol Med. 2022 Oct 5;26(21):5473–85. doi: 10.1111/jcmm.17575 (PMC9639036; doi:10.1111/jcmm.17575)
Supplement: Supplementary file 4 — Fig Legends S1‐S2 [file JCMM-26-5473-s004.docx]

Supplementary Figure 1. Altered proliferation and cycle of nasopharyngeal carcinoma cells after downregulation of EN1.
A, B CCK-8 assay detects cell proliferation capacity after downregulation of EN1. C, D Single-cell clone formation ability after downregulation of EN1 in two NPC cell lines. E, F Flow cytometric cell cycle assay detects downregulation of EN1 cell cycle changes in 5-8F and CNE-2Z cell lines. Data are shown as mean ± SD. All data from triplicate experiments.* p < 0.05, **p < 0.01, ***p < 0.001.

Supplementary Figure 2. Altered proliferation and cycle of nasopharyngeal carcinoma cells after simultaneous knockdown and overexpression EN1.
A, B CCK-8 assay detects cell proliferation capacity after simultaneous knockdown and overexpression EN1. C,D Single-cell clone formation ability after simultaneous knockdown and overexpression EN1. E, F Flow cytometric cell cycle assay detects cell cycle changes in 5-8F and CNE-2Z cell lines. G The SA-β-gal assay detects proportion of positive cells in 5-8F and CNE-2Z cell lines. H Detection of Western blotting after transfection with related viruses. Data are shown as mean ± SD. All data from triplicate experiments.* p < 0.05, **p < 0.01, ***p < 0.001.

Supplementary Table S1-S2
